# Psychiatric Influences on Hidradenitis Suppurativa: A Call for Help

**DOI:** 10.1055/a-2258-2438

**Published:** 2024-04-04

**Authors:** Holly D. Shan, Samuel S. Huffman, John D. Bovill, Zoë K. Haffner, Parhom Towfighi, Carol D. Benedict, Karen K. Evans

**Affiliations:** 1 Department of Plastic and Reconstructive Surgery, Georgetown University School of Medicine, Washington, District of Columbia; 2 Department of Plastic and Reconstructive Surgery, MedStar Georgetown University Hospital, Washington, District of Columbia; 3 Department of Rheumatology, MedStar Georgetown University Hospital, Washington, District of Columbia

**Keywords:** hidradenitis suppurativa, anxiety, depression, psychiatric comorbidities, financial stress

## Abstract

**Background** Hidradenitis suppurativa (HS) is associated with a high prevalence of psychiatric disorders. However, no studies examine how psychiatric disorders influence surgical and financial outcomes. This study aimed to assess impact of a psychiatric diagnosis on patients treated for HS.

**Methods** Patients with HS were retrospectively identified at a single institution from 2010 to 2021. Cohorts were stratified by the presence of a psychiatric disorder. Demographics, comorbidities, and disease characteristics were collected. Outcomes assessed included the procedural interventions and emergency department (ED) visits. Financial distress was assessed via the COST-FACIT Version 2 survey.

**Results** Out of 138 patients, 40 (29.0%) completed the survey of which 19 (47.5%) had a preexisting psychiatric diagnosis. No demographic differences were found between cohorts. Mean follow-up was 16.1 ± 11.0 months. The psychiatric cohort had a higher median number of surgeries received (7.0 vs. 1.5, *p* < 0.001), a higher median number of ED visits (1.0 vs. 0, *p* = 0.006), and a similar hospital length of stay ( *p* = 0.456). The mean COST-FACIT score of the overall study population was 19.2 ± 10.7 (grade 1 financial toxicity). The psych cohort had a lower mean COST-FACIT score (16.8 vs. 21.3, *p* = 0.092) and reported greater financial hardship (3.3 vs. 1.7, *p* < 0.001). On multivariate analysis, a psychiatric diagnosis was predictive of lower credit scores, more ED visits, and a higher number of surgeries.

**Conclusion** Preexisting psychiatric conditions in patients with HS are associated with increased health care utilization and surgical intervention with substantial financial distress. Plastic surgeons should be cognizant of such comorbid disorders to facilitate holistic care addressing all patient needs.

## Introduction

Hidradenitis suppurativa (HS) is considered to be one of the most life-impacting skin disorders typically occurring in young adult women with a prevalence between 1 and 4%. 1 In general, patients with skin diseases have a higher prevalence of at least one concomitant psychiatric disorder reported in up to 25% of patients, while those with HS, specifically, were found to have at least one psychiatric disorder reported in up to 57.8% compared with 7% of matched controls. 2 3 4 5 This is greater than the National Institute of Mental Health estimate that determined 21.4% of the general population of U.S. adults experience any mood disorder at some point in their life. 6 A systematic review and meta-analysis by Phan et al found that HS patients were more likely to commit suicide, have substance abuse issues than the general population, and have a higher prevalence of concomitant psychiatric diseases. 5 7

The natural course of the disease is unpredictable, characterized by flare-ups that produce painful lesions, which later become scars. 8 9 Undoubtedly, this leads to immense psychosocial impacts of HS including embarrassment, social stigma, and decreased self-esteem. 10 While similar skin conditions such as psoriasis, acne, and eczema can be controlled by medication, medical management alone for HS is often not sufficient to control symptoms of the disease. 11 HS is unique in that often surgical intervention is required for management, and the anatomic location of the disease, such as the axillary region, breast, and groin are sensitized areas that cause patient distress, shame, and embarrassment. 12 Moreover, up to 50% of HS patients receive surgery during the course of their disease, with plastic surgeons performing the majority of HS-related surgeries. 13

Although it is well-established that HS is associated with high emotional, social, mental, and monetary stress, neither study has explored how these aspects influence surgical management outcomes nor how preexisting psychiatric diseases may influence management of HS. Therefore, the study aimed to assess the impact of a preexisting psychiatric diagnosis on surgical management and patient-reported financial distress in patients with HS.

## Methods

### Retrospective Review

Patients who underwent surgical treatment for HS by senior author (K.K.E) at a single tertiary urban wound center from January 2010 to December 2021 were retrospectively reviewed. Electronic medical records were reviewed to collect patient demographics, comorbidities, disease characteristics, and treatment course. The psychiatric cohort (Psych Comorbid) was identified using Diagnostic and Statistical Manual of Mental Disorders, Fifth Edition (DSM-5) clinical diagnoses of addiction, anxiety, schizophrenia, or major depressive disorder prior to or at the time of HS diagnosis.

### Data Collection

Patient demographics incorporated variables such as patient age, sex, race, body mass index (BMI), and other medical comorbidities including smoking status and diabetes mellitus. Data regarding hidradenitis disease courses included Hurley Stage, a grading system to characterize the severity of disease in HS patients, which was assigned by an attending physician in the departments of plastic surgery, dermatology, or rheumatology. As per the grading system set by the hospital, Hurley Stage I was defined as the presence of single of multiple abscess without sinus tracts and cauterization, Stage II as recurrent single of multiple abscesses that are separated by normal skin with limited sinus tracts and cauterization, and Stage III as diffuse involvement of multiple interconnected tracks and abscesses across the entire affected area. The number of surgeries included excision, incision and drainage, complex closure, debridement, diverting colostomy, and skin graft. Complication-related surgeries were not included as they did not focus on treatment of HS specifically.

### COST Survey

The modified COST FACIT survey Version 2 is a 12-item self-reported screening tool developed by de Souza et al which has been validated to reliably demonstrate financial toxicity in cancer patients. 14 The original structure of the instrument was utilized in email-based form and given to patients for independent completion. 15 The COST survey was initially emailed to patients. If there was no survey response after 2 weeks, a reminder email was sent. If there was no survey response after the reminder email, patients were contacted via phone. Given the chronicity of the disease and high health care utilization of HS, we believed the questions proposed in this survey would resonate with our HS population, even though it is not validated outside of cancer literature.

The COST FACIT Version 2 survey employs a 12-item 5-point Likert scale format to detect the presence of financial distress present within 7 days of the survey. The 12 items on the survey are coded sequentially from 1 to 12 ( Supplementary Digital Content 1 [available in the online version only]). According to FACIT scoring guidelines, the additional item—12 is solely a summary item and must be excluded from the total score of the questionnaire. Scores are reversed for items 2, 3, 4, 5, 8, 9, and 10. Survey responses varied with values corresponding to responses—0 (not at all), 1 (a little bit), 2 (somewhat), 3 (quite a bit), and 4 (very much). The total score is summed for a maximal score of 44 with lower scores being associated with higher financial distress. Different grades of financial distress are indicated by four groups of total COST scores. No impact (Grade 0) is associated with a COST score ≥26, mild impact (Grade 1) scores are 14 to 25, moderate impact (Grade 2) scores are 1 to 13, and high impact (Grade 3) is associated with a score of 0. 16 Patients were excluded if the survey was not completed in its entirety.

Following the COST FACIT survey, a second 12-item investigator-generated survey was administered regarding treatment and socioeconomic information, such as credit score, combined household income, and employment status. Lastly, patients were asked about the presence of coping habits such as therapy, support groups, and meditation for the purposes of HS management ( Supplementary Digital Content 2 [available in the online version only]).

### Statistical Analysis

Categorical variables were reported as percentages and frequencies and assessed with univariate analyses using χ ^2^ or Fisher's exact test ( *n* < 5). Continuous variables are reported as “mean + standard deviation” and assessed using a Student's *t* -test. Potential confounders were controlled for using multivariate logistic and linear regression analyses. Variables included in our regression models included the following: credit score, average number of surgeries, hospital length of stay (LOS), and ED visits. Data analysis was performed using STATA version 17.0 (StataCorp, College Station, TX) with statistical significance set at values of *p* < 0.05.

## Results

### Patient Demographics

A total of 138 patients with HS underwent surgical treatment by plastic and reconstructive surgery and cared for by general surgery, rheumatology, urology, gynecology, and/or dermatology at our multidisciplinary wound care center. Forty patients (29.0%) were included in the study after completing the COST FACIT survey. Two patients (1.5%) did not complete the survey in its entirety and were excluded. The majority of patients were female ( *n* = 29, 21.1%). The mean age at time of initial visit was 27.1 ± 10.9 years and mean BMI was 32.9 ± 1.2 kg/m ^2^ . Median Hurley Stage of the study population was 3.0 [interquartile range 1.0] with no differences between cohorts. Mean follow-up time was 16.1 ± 11.0 months. There were 16 patients who had a history of smoking (38.10%), and 9 patients had diabetes mellitus type II (21.95%). No statistically significant demographic differences were found between cohorts ( Table 1 ).

**Table 1:** Patient demographics and general surgical management outcomes

	Total ( *n* = 40) *N* , %	Control ( *n* = 21) *N* , %	Psych comorbid ( *n* = 19) *N* , %	*p* -Value
***Demographics***				
Age (years), mean ± SD	27.1 ± 10.9	28.6 ± 11.1	25.4 ± 10.7	0.324
BMI (kg/m ^2^ ), mean ± SD	33.5 ± 1.8	32.3 ± 2.4	34.8 ± 2.6	0.487
Females	29 (72.5)	16 (80.0)	13 (72.2)	0.572
Hurley Stage [IQR]	3 [1]	3 [1]	3 [1]	0.486
Smoking Hx	16 (38.1)	6 (26.1)	10 (52.6)	0.078
Diabetes mellitus	9 (22.0)	4 (17.4)	5 (27.8)	0.428
Annual household income ($), mean ± SD	65,021.63 ± 7,981.37	71,060.67 ± 12,360.03	57,257.14 ± 9,061.58	0.392
Credit score; mean ± SD	674.2 ± 19.1	721.7 ± 62.5	600.2 ± 29.2	**<0.001**
Unemployment	14 (31.8)	5 (31.3)	9 (42.9)	0.472
Currently receiving medical treatment for HS	28 (70.0)	16 (72.7)	12 (54.6)	0.214
***Overall surgical management outcomes***				
Number of surgeries per patient; Med [IQR]	3.0 [6.0]	1.5 [3.0]	7.0 [9.0]	**<0.001**
**Surgery type**				
Any procedure	190 a	44 (23.2)	146 (76.8)	**<0.001**
Debridement	7 (3.7)	3 (6.8)	4 (2.7)	0.203
Incision and Drainage	57 (30.0)	17 (38.6)	40 (27.4)	0.154
Excision	99 (52.1)	24 (54.5)	75 (51.4)	0.482
Colostomy	15 (7.9)	0	15 (10.3)	0.088
Flap	12 (6.3)	0	12 (8.2)	0.162
Hospital length of stay (days), Med [IQR]	6 [11]	3 [4]	4 [11]	0.456
Emergency department visits, Med [IQR]	0 [3]	0 [1]	1 [12]	**0.006**
Mean follow-up (months), mean ± SD	16.1 ± 11.0	16.8 ± 12.9	15.3 ± 8.8	0.680
Adapted coping habits after HS surgery	24 (60.0)	9 (42.9)	15 (78.9)	**0.020**

Abbreviations: BMI, body mass index; HS, hidradenitits suppurativa; Hx, history; SD, standard deviation; IQR, interquartile range.

Bold *p* -values represent significance.

a Total number of procedures does not have a true percentage as it reflects the total amount of procedures for all patients in the study.

### Patient Outcomes

Of the 40 patients included, 19 (47.5%) had a confirmed diagnosis of a psychiatric disorder, which we defined as “psych comorbid,” and the 21 HS patients who did not have documented psychiatric disorders served as a control. Among the psych comorbid cohort, the diagnoses included anxiety ( *n* = 7, 36.8%), depression ( *n* = 12, 63.15%), schizophrenia ( *n* = 3, 15.8%), and addiction ( *n* = 3, 15.7%). Out of 190 surgeries, 76.84% ( *n* = 146) were performed in the psych comorbid group. In univariate logistic regression analysis, those who had a concomitant psychiatric diagnosis a higher median number of emergency department (ED) visits (1.0 vs. 0, *p* = 0.006) and higher median number of surgeries per patient (7.0 vs. 1.5, *p* < 0.0001), with similar postoperative LOS. The psych comorbid cohort also reported a greater number of adaptive coping habits (79.0 vs. 37.5%, *p* = 0.0070). Mean follow-up duration was comparable between both cohorts ( *p* = 0.680).

The overall study population COST FACIT score was 19.2 ± 10.7. Grade 0 financial toxicity was reported in 32.5% ( *n* = 13) of patients, grade 1 in 40% ( *n* = 16), and grade 2 in 30% ( *n* = 12). One patient (2.5%) reported grade 3 financial toxicity. No difference in mean COST FACIT scores were found between cohorts, although both report grade 1 financial toxicity. Upon individual analysis of each item, it was found that that the psych comorbid cohort was less satisfied with their financial situation (1.1 vs. 1.9, *p* = 0.044), felt less in control of their financial situation (1.8 vs. 2.7, *p* = 0.028), and viewed their diagnosis as a greater hardship to their family and themselves (3.3 vs. 1.6, *p* < 0.001; Table 2 ).

**Table 2:** Hidradenitits suppurativa-adjusted COST FACIT survey results

	Overall ( *n* = 40)	Control ( *n* = 21)	Psych comorbid ( *n* = 19)	*p-* Value
Survey question				
**Total score**	19.2 ± 10.7	21.3 ± 11.4	16.8 ± 9.7	0.092
FT1: I know that I have enough money in savings, retirement, or assets to cover the costs of my treatment	1.1 ± 1.5	1.4 ± 0.7	0.8 ± 1.2	0.108
FT2: My out-of-pocket medical expenses are more than I thought they would be	1.8 ± 1.5	1.7 ± 1.5	1.8 ± 1.5	0.358
FT3: I worry about the financial problems I will have in the future as a result of my illness or treatment	1.4 ± 1.5	1.4 ± 1.7	1.3 ± 1.3	0.406
FT4: I feel I have no choice about the amount of money I spend on care	1.3 ± 1.6	1.4 ± 1.7	1.1 ± 1.5	0.263
FT5: I am frustrated that I cannot work or contribute as much as I usually do	1.6 ± 1.7	2.0 ± 1.6	1.2 ± 1.7	0.059
FT6: I am satisfied with my current financial situation	1.5 ± 1.5	1.9 ± 1.6	1.1 ± 1.2	**0.044**
FT7: I am able to meet my monthly expenses	2.1 ± 1.3	2.4 ± 1.1	1.7 ± 1.5	0.064
FT8: I feel financially stressed	1.8 ± 1.5	2.1 ± 1.7	1.5 ± 1.3	0.117
FT9: I am concerned about keeping my job and income, including work at home	2.2 ± 1.6	2.3 ± 1.6	2.2 ± 1.7	0.404
FT10: My treatment has reduced my satisfaction with my present financial situation	2.2 ± 1.6	2.1 ± 1.8	2.3 ± 1.5	0.372
FT11: I feel in control of my financial situation	2.3 ± 1.4	2.7 ± 1.1	1.8 ± 1.6	**0.028**
FT12: HS has been a financial hardship to my family and me	1.1 ± 1.5	1.6 ± 1.6	3.3 ± 1.2	**<0.001**

Abbreviation: HS, hidradenitis suppurativa.

Bold *p* -values represent significance.

Scale: 0, “not at all”; 4, “very much.”

Multivariate linear regression, a concomitant psychiatric diagnosis remained a significant predictor of the number of surgeries, credit score, and number of ED visits. In patients with HS, a psychiatric diagnosis was associated with lower credit score (B = − 109.4, 95% CI −195.7 to −23.1, *p* = 0.021). Multivariate analysis identified a psychiatric diagnosis as a positive predictor of number of surgical procedures (B = 7.3, 95% CI 3.5–11.1, *p* < 0.001) and the number of ED visits (B = 4.4, 95% CI 0.7–8.2, *p* = 0.032). A concomitant psychiatric diagnosis was not associated with COST FACIT scores or hospital LOS ( Table 3 ).

**Table 3:** Multivariate linear regression of patient factors and credit score, number of hidradenitis suppurativa-related surgeries, hospital length of stay, and emergency department visits

	COST FACIT		Credit scores		Number of surgeries		Hospital LOS		ED visits	
Variable	B (95% CI)	*p* -Value	B (95% CI)	*p* -Value	B (95% CI)	*p* -Value	B (95% CI)	*p* -Value	B (95% CI)	*p* -value
Psych comorbid	7.1 (−29.1–43.3)	0.636	−109.4 (−195.7 to −23.1)	**0.021**	7.3 (3.5–11.1)	**<0.001**	−1.5 (−5.5–2.5)	0.427	4.4 (0.7–8.2)	**0.023**
Female sex	3.3 (−12.7–30.3)	0.766	53.4 (−35.7–142.6)	0.193	−2.0 (−6.9–2.9)	0.393	−6.5 (−10.9 to −2.1)	**0.007**	−2.8 (−8.2–2.6)	0.286
Hurley Stage	−1.3 (−15.8–13.3)	0.834	5.2 (−50.5–60.8)	0.828	−0.6 (−3.3–2.3)	0.688	0.3 (−2.2–2.8)	0.810	0.6 (−2.1–3.4)	0.645
Smoking Hx	0.6 (−14.6–15.8)	0.923	4.9 (−53.5–63.3)	0.843	0.5 (−2.6–3.6)	0.733	−5.2 (−8.8 to −1.6)	**0.008**	−2.6 (−5.9–0.7)	0.118
Diabetes mellitus	−6.4 (−35.1–22.3)	0.589	−14.3 (−123.7–95.1)	0.760	4.8 (−0.4–10.1)	0.070	4.0 (−1.6–9.6)	0.146	1.8 (−3.5–7.1)	0.494
Annual household income ($)	0 (0–0.1)	0.409	0 (−0.01–0)	0.843	–	–	–	–	–	–
Credit score	−0.02 (−0.3–0.2)	0.818	–	–	–	–	–	–	–	–

Abbreviations: ED, emergency department; Hx, history; LOS, length of stay; CI, confidence interval.

## Discussion

HS can leave a devastating physical, mental, and financial toll on a patient's well-being. As such, this is the first study to explore the interplay between preexisting psychiatric conditions and surgical treatment, frequency of ED visits, and patient-reported financial distress in those with HS. The psych comorbid group had a significantly higher number of surgeries and ED visits related to HS and endured greater financial difficulties in several aspects outlined in the COST FACIT survey.

Prevalence of psychiatric disorders in the cohort of patients who completed COST FACIT surveys was 45.5%, which is comparable to Shlyankevich et al who reported a prevalence of 56% in HS patients at a single tertiary care center comparable to our institution. 5 This is substantially higher than the lifetime prevalence of anxiety (28.8%) and mood disorders (20.8%) found in the 9,282 respondents of the National Comorbidity Survey Replication. 17 Although the etiology of HS is unclear, it is understood that the risk factors for onset of the disease include smoking, obesity, sex, and age. 18 19 In our cohort, both groups were found to have no differences in these risk factors suggesting there are other underlying reasons for the greater surgical intervention and financial impact. One hypothesis is that psychiatric illness exacerbates inflammation leading to a worse disease status. Mental illnesses such as depression, anxiety, and bipolar disorder are often associated with a chronic low-grade inflammatory response which activates cell-mediated immunity and the compensatory anti-inflammatory reflex system. 20 These disorders are also accompanied by increased oxidative and nitrative stress, leaving the body in a vulnerable state that can potentiate chronic inflammatory diseases like HS. 21

Several treatment strategies exist for HS, dependent on stage and anatomic location of disease. When surgical intervention is required, procedures include but are not limited to debridement, incision and drainage, limited and wide excisions, diverting colostomies for perianal HS, and local and free flap reconstruction. 22 23 24 25 Our results demonstrate psych comorbid patients received almost five times more surgeries than the control (7.0 vs. 1.5 surgeries) and was independently predictive of increased number of operations for treatment of HS despite the similar Hurley staging across both groups. This is likely related to a proportional relationship between psychiatric disease and HS presentation in that patients with psychiatric disease associated with HS might present later in the course of their disease and be prone to less complaining behavior. 26 27 As surgical treatment for patients with chronic diseases can induce states of extreme stress, and the worsened HS disease state potentiates the psychiatric disease and thereby creating a negative feedback loop. 28 For those with limited health insurance coverage or those who cannot afford routine care or targeted therapies such as adalimumab, their disease may progress, requiring repeat procedures that confer even greater financial cost. 29 30

In this study, we also found that a concomitant psychiatric disease in patients with HS was not associated with longer hospital LOS. Regardless, prolonged hospital stays can lead to worsening of psychiatric disease and should be avoided for those with known diagnoses. Kitagawa et al reported that depression was associated with a greater hospital LOS for patients undergoing thoracic surgery, but the prevalence of depression was underestimated as the study reported some patients were uncomfortable confiding in their surgeons about mental health. 31 In addition, each day in the hospital increases the risk for adverse events such as an adverse drug reaction, ulcers, and infection. 32 Recognizing the potential risks of prolonged hospital LOS on patients with a concomitant psychiatric diagnosis, offers an area of improvement regarding mental health screenings and patient management.

In our cohort, a psychiatric disease in patients with HS was associated with 4.4 more ED visits when controlling for other factors known to influence HS disease severity and flares. It has been shown that patients with HS utilize high-cost settings, such as the ED, more often than the general population, which may further decrease the financial outlook of this disease. 33 Khalsa et al reported that HS patients had an average of 4.8 ED claims. 34 Additionally, the unpredictable and painful nature of flare-ups for HS often lead to ED admissions. In 2021, the average annual cost for ED visits for HS treatment was over 11 million dollars and the average cost of care per ED visits excluding inpatient costs was over $600 which is greater than average spent on ED visits for other skin and subcutaneous diseases. 34

The chronicity of HS leads to a substantial financial commitment that is compounded by immense psychosocial considerations. Estimated costs for a patient with HS range around $7,000 every 3 years to over $23,000 for 5 years. 33 34 As such, the overall study population reported grade 1 financial toxicity at comparable levels to cancer populations suggesting that HS is financially debilitating. 35 36 37 38 Moreover, credit scores are used to assess socioeconomic factors that contribute to patient health outcomes and demonstrate financial consequences of disease events. 39 40 41 42 The psych comorbid cohort reported 121 points lower mean credit score than those without comorbid psychiatric illness, and presence of the comorbidity was a significant predictor of lower credit scores. Moreover, the psych comorbid group reported significantly lower satisfaction and less control over their financial situation, as well as viewed their diagnosis as a greater burden to their family. Compared with cross-matched controls, Tzellos et al found that patients newly diagnosed with HS were reported to have slower income growth, high risk of leaving the workforce, and have more total days of work lost. 43 Interestingly, our cohort of psych comorbid patients had similar rates of unemployment even though they reported more concern about finances, thus psychiatric disease may have negatively influenced the financial outlook of the psych comorbid group.

The study is limited by its retrospective study design, which relied on the quality and accuracy of electronic medical records. Additionally, the prevalence of a psychiatric disorder was determined from patient reports or a DSM-5 diagnosis available within the patient's problem list at the time of HS diagnosis, and therefore, may be underreported, as patients may not have disclosed their illness. The study may also be limited by selection bias in that the patients included in the study were able to seek treatment and may have more financial ability than patients who have HS but did not choose to seek care. As such this may be one explanation as to why we did not find differences in self-reported finances of our two cohorts. Further multi-institutional studies are required to determine the full extent of the impact of psychiatric diagnoses on the outcomes of patients with concomitant HS.

### Conclusion

Preexisting psychiatric conditions in patients with HS are associated with increased health care utilization, surgical intervention, and financial distress. Plastic surgeons should be cognizant of such comorbid disorders to facilitate holistic care addressing all patient needs.

## Supplementary Material

Supplementary Digital Content 1 Supplementary Digital Content 1

Supplementary Digital Content 2 Supplementary Digital Content 2

## References

[1] Quality of Life Group of the French Society of Dermatology Wolkenstein P Loundou A Barrau K Auquier P Revuz J Quality of life impairment in hidradenitis suppurativa: a study of 61 cases J Am Acad Dermatol 2007 56 04 621 623 17097366 10.1016/j.jaad.2006.08.061

[2] Dalgard F J Gieler U Tomas-Aragones L The psychological burden of skin diseases: a cross-sectional multicenter study among dermatological out-patients in 13 European countries J Invest Dermatol 2015 135 04 984 991 25521458 10.1038/jid.2014.530 PMC4378256

[3] Hughes J E Barraclough B M Hamblin L G White J E Psychiatric symptoms in dermatology patients Br J Psychiatry 1983 143 01 51 54 6882992 10.1192/bjp.143.1.51

[4] Picardi A Abeni D Melchi C F Puddu P Pasquini P Psychiatric morbidity in dermatological outpatients: an issue to be recognized Br J Dermatol 2000 143 05 983 991 11069507 10.1046/j.1365-2133.2000.03831.x

[5] Shlyankevich J Chen A J Kim G E Kimball A B Hidradenitis suppurativa is a systemic disease with substantial comorbidity burden: a chart-verified case-control analysis J Am Acad Dermatol 2014 71 06 1144 1150 25440440 10.1016/j.jaad.2014.09.012

[6] National Institute of Mental Health.Any Mood DisorderAccessed December 19, 2022 at:https://www.nimh.nih.gov/health/statistics/any-mood-disorder

[7] Phan K Huo Y R Smith S D Hidradenitis suppurativa and psychiatric comorbidities, suicides and substance abuse: systematic review and meta-analysis Ann Transl Med 2020 8 13 821 821 32793666 10.21037/atm-20-1028 PMC7396254

[8] Gooderham M Papp K The psychosocial impact of hidradenitis suppurativa J Am Acad Dermatol 2015 73(5 Suppl 1)S19 S22 26470609 10.1016/j.jaad.2015.07.054

[9] Matusiak L Bieniek A Szepietowski J C Psychophysical aspects of hidradenitis suppurativa Acta Derm Venereol 2010 90 03 264 268 20526543 10.2340/00015555-0866

[10] Esmann S Jemec G BE Psychosocial impact of hidradenitis suppurativa: a qualitative study Acta Derm Venereol 2011 91 03 328 332 21394419 10.2340/00015555-1082

[11] Katzman J H Tahmasbi M Ghayouri M Nanjappa S Li M C Greene J Management of severe hidradenitis suppurativa Cureus 2021 13 02 e13483 e13483 33777570 10.7759/cureus.13483 PMC7989970

[12] Koumaki D Efthymiou O Bozi E Katoulis A C Perspectives on perceived stigma and self-stigma in patients with hidradenitis suppurativa Clin Cosmet Investig Dermatol 2019 12 785 790 10.2147/CCID.S180036 PMC6801565 31802927

[13] Garbayo-Salmons P Romaní J Ferrer de la Fuente C Pallisera Lloveras A López-LLunell C Prat Escayola J Hidradenitis suppurative: our experience with a surgical case management team Actas Dermosifiliogr (Engl Ed) 2020 111 05 408 412(English Edition) 32408974 10.1016/j.ad.2019.12.001

[14] de Souza J A Yap B J Wroblewski K Measuring financial toxicity as a clinically relevant patient-reported outcome: the validation of the COmprehensive Score for financial Toxicity (COST) Cancer 2017 123 03 476 484 27716900 10.1002/cncr.30369 PMC5298039

[15] Webster K Cella D Yost K The Functional Assessment of Chronic Illness Therapy (FACIT) Measurement System: properties, applications, and interpretation Health Qual Life Outcomes 2003 1 79 14678568 10.1186/1477-7525-1-79 PMC317391

[16] Dar M A Chauhan R Sharma K K Trivedi V Dhingra S Murti K Assessing the reliability and validity of comprehensive score for financial toxicity (COST) among radiation oncology patients in India: a cross-sectional pilot study Ecancermedicalscience 2021 15 1219 34158823 10.3332/ecancer.2021.1219 PMC8183655

[17] Kessler R C Berglund P Demler O Jin R Merikangas K R Walters E E Lifetime prevalence and age-of-onset distributions of DSM-IV disorders in the National Comorbidity Survey Replication Arch Gen Psychiatry 2005 62 06 593 602 15939837 10.1001/archpsyc.62.6.593

[18] Alikhan A Sayed C Alavi A North American clinical management guidelines for hidradenitis suppurativa: A publication from the United States and Canadian Hidradenitis Suppurativa Foundations: Part I: Diagnosis, evaluation, and the use of complementary and procedural management J Am Acad Dermatol 2019 81 01 76 90 30872156 10.1016/j.jaad.2019.02.067 PMC9131894

[19] Ingram J R Hidradenitis suppurativa: pathogenesis, clinical features, and diagnosisUpToDate. Accessed September 11, 2022 at:https://www.uptodate.com/contents/hidradenitis-suppurativa-pathogenesis-clinical-features-and-diagnosis?search=hidradenitis-suppu-&topicRef=110073&source=see_link

[20] Maes M Evidence for an immune response in major depression: a review and hypothesis Prog Neuropsychopharmacol Biol Psychiatry 1995 19 01 11 38 7708925 10.1016/0278-5846(94)00101-m

[21] Maes M Berk M Goehler L Depression and sickness behavior are Janus-faced responses to shared inflammatory pathways BMC Med 2012 10 01 66 22747645 10.1186/1741-7015-10-66 PMC3391987

[22] Wollina U Koch A Heinig B Kittner T Nowak A Acne inversa (Hidradenitis suppurativa): A review with a focus on pathogenesis and treatment Indian Dermatol Online J 2013 4 01 2 11 23439959 10.4103/2229-5178.105454 PMC3573446

[23] Ching D L Mughal M Papas A Soldin M Axillary reconstruction for hidradenitis suppurativa with an inner-arm transposition flap creating a brachioplasty effect Arch Plast Surg 2017 44 03 228 233 28573098 10.5999/aps.2017.44.3.228 PMC5447533

[24] Bocchini S F Habr-Gama A Kiss D R Imperiale A R Araujo S EA Gluteal and perianal hidradenitis suppurativa: surgical treatment by wide excision Dis Colon Rectum 2003 46 07 944 949 12847371 10.1007/s10350-004-6691-1

[25] Jemec G BE Clinical practice. Hidradenitis suppurativa N Engl J Med 2012 366 02 158 164 22236226 10.1056/NEJMcp1014163

[26] Leiphart P Kitts S Sciacca Kirby J Adherence to over-the-counter antimicrobial washes in hidradenitis suppurativa patients Dermatology 2019 235 05 440 441 31242490 10.1159/000500827

[27] Kokolakis G Wolk K Schneider-Burrus S Delayed diagnosis of hidradenitis suppurativa and its effect on patients and healthcare system Dermatology 2020 236 05 421 430 32610312 10.1159/000508787 PMC7592906

[28] Neeman E Ben-Eliyahu S Surgery and stress promote cancer metastasis: new outlooks on perioperative mediating mechanisms and immune involvement Brain Behav Immun 2013 30 S32 S40 22504092 10.1016/j.bbi.2012.03.006 PMC3423506

[29] Frings V G Schöffski O Goebeler M Presser D Economic analysis of the costs associated with Hidradenitis suppurativa at a German University Hospital PLoS ONE 2021 16 08 e0255560 34347845 10.1371/journal.pone.0255560 PMC8336817

[30] Tsentemeidou A Sotiriou E Ioannides D Vakirlis E Hidradenitis suppurativa-related expenditure, a call for awareness: systematic review of literature J Dtsch Dermatol Ges 2022 20 08 1061 1072 10.1111/ddg.14796 35821567

[31] Kitagawa R Yasui-Furukori N Tsushima T Kaneko S Fukuda I Depression increases the length of hospitalization for patients undergoing thoracic surgery: a preliminary study Psychosomatics 2011 52 05 428 432 21907061 10.1016/j.psym.2011.03.010

[32] Hauck K Zhao X How dangerous is a day in hospital? A model of adverse events and length of stay for medical inpatients Med Care 2011 49 12 1068 1075 21945976 10.1097/MLR.0b013e31822efb09

[33] Kirby J S Miller J J Adams D R Leslie D Health care utilization patterns and costs for patients with hidradenitis suppurativa JAMA Dermatol 2014 150 09 937 944 24908260 10.1001/jamadermatol.2014.691

[34] Khalsa A Liu G Kirby J S Increased utilization of emergency department and inpatient care by patients with hidradenitis suppurativa J Am Acad Dermatol 2015 73 04 609 614 26190241 10.1016/j.jaad.2015.06.053

[35] Bouberhan S Shea M Kennedy A Financial toxicity in gynecologic oncology Gynecol Oncol 2019 154 01 8 12 31053404 10.1016/j.ygyno.2019.04.003 PMC7001853

[36] Huntington S F Weiss B M Vogl D T Financial toxicity in insured patients with multiple myeloma: a cross-sectional pilot study Lancet Haematol 2015 2 10 e408 e416 26686042 10.1016/S2352-3026(15)00151-9

[37] Hazell S Z Fu W Hu C Financial toxicity in lung cancer: an assessment of magnitude, perception, and impact on quality of life Ann Oncol 2020 31 01 96 102 31912803 10.1016/j.annonc.2019.10.006

[38] D'Rummo K A Miller L TenNapel M J Shen X Assessing the financial toxicity of radiation oncology patients using the validated comprehensive score for financial toxicity as a patient-reported outcome Pract Radiat Oncol 2020 10 05 e322 e329 31634632 10.1016/j.prro.2019.10.005

[39] Houle J N Collins J M Schmeiser M D Flu and finances: influenza outbreaks and loan defaults in US Cities, 2004-2012 Am J Public Health 2015 105 09 e75 e80 10.2105/AJPH.2015.302671 PMC4539813 26180971

[40] Li Y Gao J Enkavi A Z Zaval L Weber E U Johnson E J Sound credit scores and financial decisions despite cognitive aging Proc Natl Acad Sci U S A 2015 112 01 65 69 25535381 10.1073/pnas.1413570112 PMC4291658

[41] Israel S Caspi A Belsky D W Credit scores, cardiovascular disease risk, and human capital Proc Natl Acad Sci U S A 2014 111 48 17087 17092 25404329 10.1073/pnas.1409794111 PMC4260546

[42] Dean L T Schmitz K H Frick K D Consumer credit as a novel marker for economic burden and health after cancer in a diverse population of breast cancer survivors in the USA J Cancer Surviv 2018 12 03 306 315 29372485 10.1007/s11764-017-0669-1 PMC5955811

[43] Tzellos T Yang H Mu F Calimlim B Signorovitch J Impact of hidradenitis suppurativa on work loss, indirect costs and income Br J Dermatol 2019 181 01 147 154 30120887 10.1111/bjd.17101 PMC7379487

