# Supplementary material for: Psychiatric Influences on Hidradenitis Suppurativa: A Call for Help
Source: Arch Plast Surg. 2024 Apr 4;51(3):304–10. doi: 10.1055/a-2258-2438 (PMC11081730; doi:10.1055/a-2258-2438)
Supplement: Supplementary file 1 — Supplementary Digital Content 1 [file 10-1055-a-2258-2438-s22dec0226oa-1.pdf]

Supplementary Digital Content 1 Cost – FACIT (Version 2) survey items.

Below is a list of statements that other people with your illness have said are important. **Please circle or mark one number per line to indicate your response as it applies to the past 7 days.**

		Not at all	A little bit	Some-what	Quite a bit	Very much
FT1	I know that I have enough money in savings, retirement, or assets to cover the costs of my treatment	0	1	2	3	4
FT2	My out-of-pocket medical expenses are more than I thought they would be	0	1	2	3	4
FT3	I worry about the financial problems I will have in the future as a result of my illness or treatment	0	1	2	3	4
FT4	I feel I have no choice about the amount of money I spend on care	0	1	2	3	4
FT5	I am frustrated that I cannot work or contribute as much as I usually do	0	1	2	3	4
FT6	I am satisfied with my current financial situation	0	1	2	3	4
FT7	I am able to meet my monthly expenses	0	1	2	3	4
FT8	I feel financially stressed	0	1	2	3	4
FT9	I am concerned about keeping my job and income, including work at home	0	1	2	3	4
FT10	My cancer or treatment has reduced my satisfaction with my present financial situation	0	1	2	3	4
FT11	I feel in control of my financial situation	0	1	2	3	4
FT12	My illness has been a financial hardship to my family and me	0	1	2	3	4

Copyright 2014, FACIT and The University of Chicago
