# Supplementary material for: Psychiatric Influences on Hidradenitis Suppurativa: A Call for Help
Source: Arch Plast Surg. 2024 Apr 4;51(3):304–10. doi: 10.1055/a-2258-2438 (PMC11081730; doi:10.1055/a-2258-2438)
Supplement: Supplementary file 2 — Supplementary Digital Content 2 [file 10-1055-a-2258-2438-s22dec0226oa-2.pdf]

Supplementary Digital Content 2 Investigator-generated survey items

Survey item number	Survey questions
1	What is your current employment status—full time, part time, not currently employed, retired?
2	What is your most recent credit score?
3	What is your annual household income by all residents?
4	After undergoing surgery to treat your hidradenitis suppurativa, did you engage in adaptive coping habits such as therapy, support groups, and meditation for the purposes of hidradenitis suppurativa management?

 © 2024. The Author(s).
